# Posterior Fossa Decompression Versus Syringo-Subarachnoid Shunt for Chiari I-associated Syringomyelia: A Systematic Review

**DOI:** 10.7759/cureus.99276

**Published:** 2025-12-15

**Authors:** Ali Alzain, Saeed Halabieh, Wejdan Hamoud, Faris Almanea, Renad Alrdeeni, Ali Bin Yameen, Ghana Azraq, Hanan Basabeen, Anmar Zabidi, Mazin Hariri, Meshari Alzahrani, Ziad Alzahrani, Taghreed A Alsinani

**Affiliations:** 1 Neurological Surgery, Ibn Sina National College, Jeddah, SAU; 2 Neurosurgery, Ibn Sina National College, Jeddah, SAU; 3 Surgery, Ibn Sina National College, Jeddah, SAU; 4 Internal Medicine, Ibn Sina National College, Jeddah, SAU; 5 Neurological Surgery, College of King Abdulaziz University, Rabigh, Jeddah, SAU; 6 General Medicine, Ibn Sina National College for Medical Studies, Jeddah, SAU; 7 Neurological Surgery, Ibn Sina National College for Medical Studies, Jeddah, SAU; 8 General Medicine, Ibn Sina National College, Jeddah, SAU; 9 Neurological Surgery, King Faisal Specialist Hospital and Research Centre, Jeddah, SAU; 10 Department of Neurosurgery, King Fahad General Hospital, Jeddah, SAU; 11 Department of Surgery, King Fahad General Hospital, Jeddah, SAU

**Keywords:** chiari malformation type i, duraplasty, posterior fossa decompression, syringomyelia, syringo-subarachnoid shunt, systematic review

## Abstract

Chiari malformation type I (CM-I) is a downward herniation of the cerebellar tonsils through the foramen magnum, frequently resulting in syringomyelia, which affects up to 75% of patients and remains a major source of neurological morbidity. This systematic review evaluated surgical outcomes for patients with CM-I-associated syringomyelia. Fifteen studies met the inclusion criteria, and the majority demonstrated high methodological quality. Posterior fossa decompression with duraplasty achieved approximately 80% syrinx resolution and 60-100% clinical improvement. Arachnoid-preserving techniques reduced CSF-related complications. Syringo-subarachnoid shunting provided 70-85% syrinx reduction after failed decompression but showed variable long-term outcomes. Posterior fossa decompression with duraplasty remains the most effective and durable treatment, while shunting is reserved for persistent or recurrent cases following the decompression.

## Introduction and background

Chiari malformation type I (CM-I) is a structural disorder characterized by the downward displacement of the cerebellar tonsils through the foramen magnum or craniocervical junction (CCJ). It can be observed in both children and adults [1-4]. This condition was first described in 1891 by the Austrian pathologist Hans Chiari [3]. CM-I is frequently complicated by syringomyelia (SM), which affects approximately 75% of patients and can produce a wide range of symptoms [4]. Common clinical manifestations include occipital headaches triggered by coughing, dysesthesia, hypoesthesia, muscle weakness, gait ataxia, and, if the syrinx extends into the brainstem, swallowing difficulties [3]. The exact pathophysiological mechanisms leading to SM in CM-I remain unclear and controversial [3-5]. Two theories for SM formation exist: (1) water-hammer theory and (2) one-way valve theory. The first suggests that arterial pulsations from the choroid plexus drive cerebrospinal fluid (CSF) downward through an abnormal fourth ventricle, contributing to syrinx formation. The second theory proposes that uneven pressures generated during Valsalva maneuvers increase spinal cord pressure, promoting syringomyelia development. Although neither hypothesis is fully established, MRI studies have identified abnormal ventricle-to-ventricle connections in CM-I patients [6-8]. Thus, the primary goal of treatment is to relieve compression on the brainstem and spinal cord by increasing the volume of the posterior fossa, thereby restoring normal CSF circulation [6]. In patients with CM-I and associated syringomyelia, surgical intervention aims to reduce tonsillar pressure on the cervicomedullary junction and normalize CSF flow across the foramen magnum. Common approaches include posterior fossa decompression (PFD) with duraplasty and arachnoid preservation, as well as shunting procedures such as syringopleural or syringo-subarachnoid shunts (SSS) [7-9]. Interestingly, in asymptomatic individuals, the presence of a syrinx alone may justify surgery, although careful observation is also appropriate. Once symptoms appear, surgical intervention is generally recommended. Notably, PFD has a high success rate in restoring CSF flow and reducing posterior fossa crowding; however, approximately 25% of syrinxes may persist. Most patients experience syrinx reduction within the first year after surgery. In cases where decompression alone is insufficient, a second PFD or shunt procedure may be performed. Long-term studies indicate that direct shunting can be challenging, with up to 50% of patients requiring reoperation [10].

Therefore, this systematic review aims to compare the clinical outcomes, complication rates, and long-term effectiveness of PFD surgery and SSS placement in the treatment of CM-I-associated syringomyelia.

## Review

Methodology

**Figure 1:**
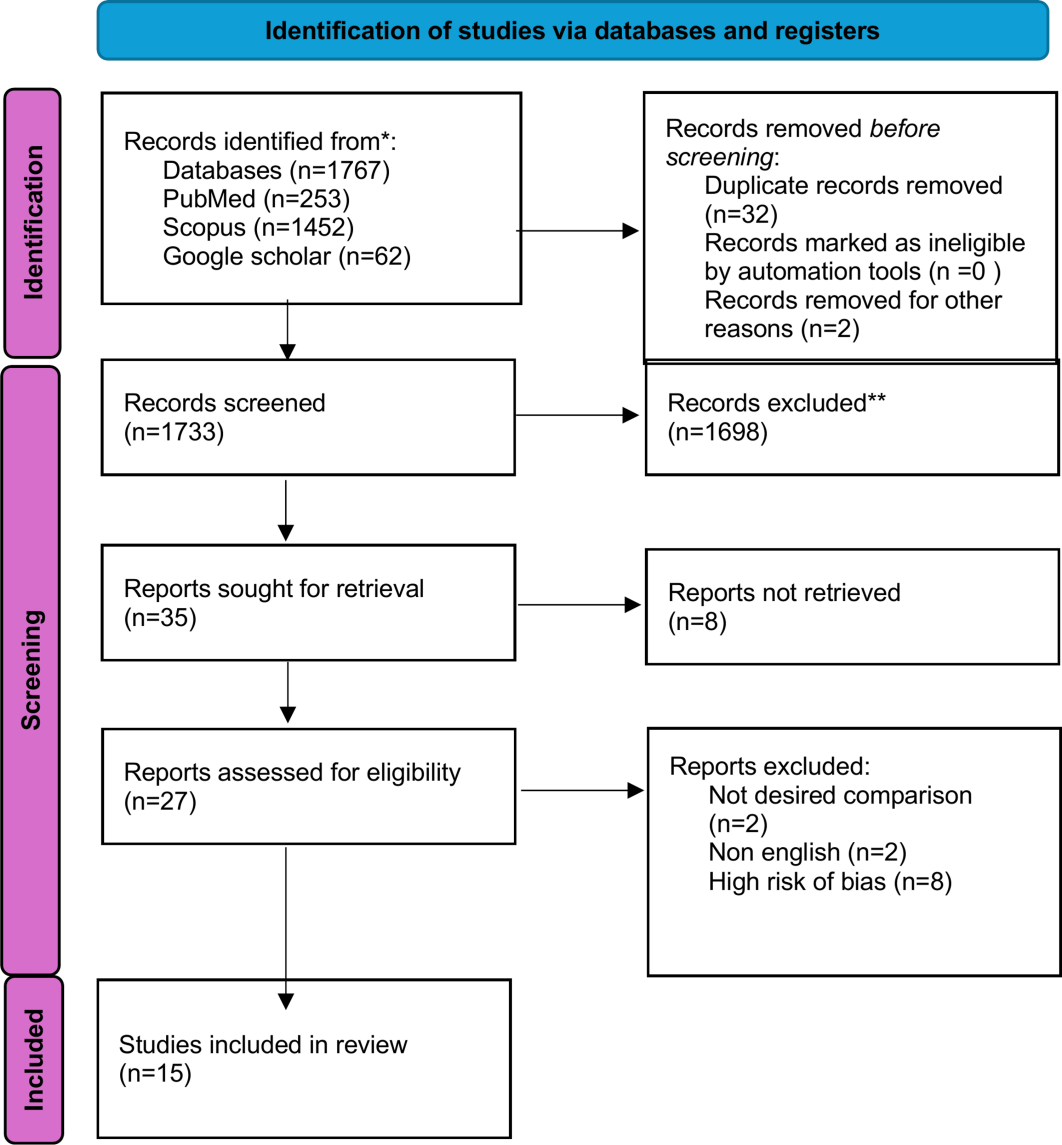
PRISMA flowchart

All studies were screened based on title and abstract using Rayyan software, investigating the comparison of the PFD versus SSS for Chiari I-associated syringomyelia. Our search encompassed multiple databases, including PubMed, Scopus, and Google Scholar, to compile a comprehensive dataset on the neurological outcome of PFD (Posterior fossa decompression) and SSAC (syringo-subarachnoid shunt). The following texts were used for the advanced searching process: (“Chiari I malformation” OR “Chiari type I” OR “Chiari malformation” OR “syringomyelia” OR “syrinx”) AND (“posterior fossa decompression” OR “suboccipital decompression” OR “foramen magnum decompression” OR “duraplasty” OR “shunt*” OR “surgical treatment” OR “surgery”) AND (“syrinx resolution” OR “syrinx reduction” OR “symptom relief” OR “neurological improvement” OR “complications” OR “clinical outcomes” OR “efficacy” OR “safety” OR “outcomes”) AND (“systematic review” OR “meta-analysis” OR “clinical study” OR “retrospective study” OR “prospective study”). Cross-sectional, case reports, randomized control trials, non-randomized controlled trials, case series, and systematic review studies published between January 2000 and July 2025 were included if the following criteria were fulfilled: 1) patients underwent PFD or SSS placement. 2) reported outcomes of any of the following (headache, blurry vision, shunt malfunction or failure, CSF over drainage, surgical site complications like pseudomeningocele formation and wound dehiscence, persistence or recurrence of syringomyelia, neurological symptoms deterioration like motor or sensory deficits, brainstem compression). Studies not published in English, published outside the inclusion data range, procedures other than PFD and SSS placement, or those with improper methods (animal studies) were excluded. In total, 1767 studies were identified and screened based on their title and abstract by 4 independent reviewers and a fifth resolving any disagreements. Thirty-two duplicates were excluded. The remaining 1733 were screened. Of these, 35 were initially included and assessed for eligibility, of which 27 met the inclusion criteria. A total of 17 were excluded after full-text reading (n=2, Not desired comparison, n=2 Non-English, n=8, High risk of bias). Selection results are summarized in the PRISMA‑2020 flow diagram (Figure 1).

Data Extraction and Quality Assessment

During the review process, full texts were included for further analysis only after achieving unanimous agreement among team members regarding their relevance and quality. Variable facilitating data extraction included research details (authors, publication year, title) and methodological aspects (study design, sample size, gender, preoperative symptoms, type of intervention, concomitant procedure, follow-up duration, syrinx resolution, symptomatic relief, complication, long-term effectiveness, radiological outcome).

Risk of Bias Assessment

We used the Newcastle-Ottawa Scale, which shows 11 out of 15 low risk of bias, while the remaining four articles showed moderate risk of bias, and this enhanced the validity and reliability of this systematic review.

Results

From 1,767 database records (PubMed = 253, Scopus = 1,452, Google Scholar = 62), thirty-two duplicates were removed, leaving 1,733 records for screening. Thirty-five full-text reports were retrieved and assessed for eligibility, and 15 studies were included in the final review (Figure 1). Exclusions were due to undesired comparisons (n = 2), non-English publications (n = 2), or high risk of bias (n = 8) (Figure 1).

Across the 15 included studies, most were retrospective cohorts or case series, while only two were prospective in design. The studies collectively included both pediatric and adult populations, with follow-up durations ranging from six months to over 10 years. Sample sizes varied from small, single-center cohorts to a large pediatric population of 245 participants, reflecting diversity in surgical technique and clinical context [1-15]. The primary surgical interventions included PFD, performed either with or without duraplasty. Several studies applied arachnoid-preserving techniques or compared extradural and intradural methods. Others evaluated selective tonsillar management or shunting procedures in patients with persistent or recurrent syringomyelia. Some reports also described adjunctive spinal fusion or CSF diversion when clinically indicated [1-10]. Radiological improvement of the syrinx was reported in most studies after decompression. Several investigations demonstrated complete or near-complete syrinx collapse, while partial reduction was also common depending on technique and duration of follow-up [1-9]. Studies employing arachnoid-preserving duraplasty consistently achieved marked syrinx reduction with fewer CSF-related complications [1-2]. Posterior fossa decompression with duraplasty (PFDD) generally produced better results than bone-only decompression (PFD) in both syrinx resolution and clinical improvement. However, smaller craniotomies sometimes achieved similar efficacy with fewer postoperative complications [2-10]. Clinical outcomes largely mirrored radiological findings. Between 60% and 100% of patients experienced symptomatic improvement, most notably headache relief and sensory or motor recovery after decompression [1, 3, 4, 7]. Functional gains were generally sustained during long-term follow-up, although some studies noted that MRI changes did not always correlate directly with clinical recovery [3-4]. In pediatric cohorts with scoliosis, early decompression often stabilized or improved spinal curvature in approximately two-thirds of patients. For those with persistent syringomyelia following decompression, shunting procedures achieved additional symptom relief and further syrinx reduction [8, 10, 12, 15]. When comparing PFDD and bone-only PFD, PFDD consistently demonstrated higher rates of syrinx resolution and clinical improvement, albeit with a slightly greater incidence of CSF-related complications [2, 6, 8, 10]. Studies evaluating craniotomy size found similar effectiveness between smaller and larger decompressions, with lower morbidity in smaller procedures. One report also indicated that extradural decompression was associated with reduced reoperation rates compared to intradural techniques, though the long-term revision burden remained high [5, 7, 9]. Selective tonsillar coagulation or limited resection did not consistently improve outcomes. Multiple studies emphasized that adequate bony decompression and restoration of CSF flow were the main goals, rendering additional tonsillar procedures unnecessary when anatomical correction was achieved [3, 4, 6]. CSF leakage and pseudomeningocele were the most frequent complications, primarily among duraplasty cohorts. Their rates were generally low, ranging from single-digit to low-teen percentages. Postoperative infections and hydrocephalus were uncommon, and most complications were managed successfully without lasting deficits [1, 2, 5, 7]. Reoperation rates varied widely depending on surgical approach and population. In pediatric series involving extradural or intradural decompression, reoperation occurred in up to half of cases over long-term follow-up in one study, whereas larger PFDD cohorts demonstrated relatively low revision rates. When revision surgery was performed for persistent obstruction, it typically led to further syrinx reduction and symptomatic improvement [8, 10, 12-15]. Long-term follow-up demonstrated durable decompression and sustained symptom relief, with high patient satisfaction. Surgical failures were most often related to incomplete restoration of CSF flow or postoperative scarring, both of which improved after revision when indicated [1, 3, 7, 9]. In studies focusing on shunting after failed decompression, newer technical refinements such as ventral shunt placement were associated with fewer malfunctions and durable syrinx reduction with corresponding clinical improvement [10, 12]. Heterogeneity among studies stemmed from differences in patient demographics, surgical strategies, adjunctive procedures, and follow-up duration, limiting data pooling for meta-analysis. Nevertheless, the overall trend was consistent: PFDD achieved superior outcomes to bone-only PFD, and arachnoid preservation contributed to sustained CSF flow normalization [2, 5-9]. Pediatric cohorts more frequently required revision or spinal fusion, particularly with associated scoliosis, while adult groups reported CSF-related complications as the principal trade-off of more invasive intradural approaches [3-6, 13-15]. Across all studies, syrinx improvement or complete resolution following PFDD or foramen magnum decompression ranged from the high-70% range to nearly complete resolution in some cohorts. PFDD consistently yielded higher rates of clinical and radiological improvement than bone-only decompression. CSF leaks were rare, pseudomeningocele even rarer, and extradural decompression was occasionally associated with reduced failure and reoperation rates despite long-term variability [1, 2, 5-10, 13-14]. The included studies are summarized in Table 1.

**Table 1:** Summary of the studies included on Chiari I-associated syringomyelia CCOS: Chicago Chiari Outcome Scale

Study (et al.)	Objective	Study Design	Sample Size	Main Findings	Quality (NOS)
Turk et al., 2025	To evaluate outcomes of arachnoid-preserving technique in Chiari I ± syringomyelia	Retrospective observational cohort	89	Arachnoid-preserving PFDD achieved significant syrinx reduction and symptom improvement with minimal CSF complications.	8/9 – Low risk
Vakharia et al., 2012	To compare outcomes in syrinx vs non-syrinx Chiari malformations after decompression	Prospective cohort	67	PFDD provided superior radiologic and clinical improvement compared with bone-only decompression; long-term efficacy maintained.	8/9 – Low risk
Kumar et al., 2018	To correlate syrinx reduction with functional outcomes after PFD ± duraplasty	Prospective cohort	22	PFDD achieved greater syrinx reduction and CCOS score improvement; collapse correlated with symptom relief.	8/9 – Low risk
Kemerdere et al., 2020	To assess clinical and radiological results of arachnoid-preserving decompression	Retrospective cohort	48	Marked and sustained clinical improvement; 80% syrinx resolution; low complication rate.	8/9 – Low risk
Arora et al., 2004	To determine predictive value of radionuclide cisternography for surgical outcome	Prospective cohort	17	Positive cisternography flow predicted syrinx reduction and better postoperative outcomes.	8/9 – Low risk
Zhang et al., 2011	To compare efficacy of various decompression techniques	Retrospective cohort	60	PFDD superior to bone-only FMD; 85% syrinx collapse; low CSF-related morbidity.	8/9 – Low risk
Akbari et al., 2022	To compare PFDD vs PFD in pediatric Chiari I with syringomyelia	Multicenter (retrospective + prospective) cohort	692	PFDD resulted in higher rates of syrinx and symptom improvement, though with slightly increased CSF events.	8/9 – Low risk
Chae and Greenfield, 2021	To analyze outcomes and predictors of revision Chiari surgery in young children	Retrospective pediatric cohort	40	Reoperation improved symptoms in ~70%; early revision associated with better syrinx resolution.	8/9 – Low risk
Verhofste et al., 2020	To evaluate long-term outcomes of decompression in CM-I with scoliosis	Retrospective case series	65	78% syrinx improvement; scoliosis stabilization; excellent long-term outcomes.	8/9 – Low risk
Soleman et al., 2019	To identify causes of decompression failure and management strategies	Retrospective cohort	48	25% required revision; extradural techniques had fewer failures; reoperation effective for persistent obstruction.	8/9 – Low risk
Tosi et al., 2020	To investigate persistent syringomyelia after PFDD	Retrospective case series	30	Persistent syrinx in ~30%; re-decompression improved flow and symptoms.	7/9 – Moderate
Soleman et al., 2019	To evaluate syringo-subarachnoid shunt (SSS) for persistent syrinx after decompression	Retrospective case series	21	80% syrinx reduction; significant clinical improvement; safe salvage approach.	7/9 – Moderate
Park et al., 2009	To identify factors influencing syrinx improvement after surgery	Retrospective comparative cohort	57	Adequate decompression and preserved arachnoid flow associated with best radiologic outcomes.	8/9 – Low risk
Tubbs et al., 2004	To describe persistent syrinx after pediatric decompression	Retrospective case series	8	Persistent syrinx due to arachnoid scarring; reoperation with stent improved drainage.	6/9 – Moderate

Discussion

This systematic review evaluated and compared the outcomes of PFD and SSS in patients with Chiari malformation type I (CM-I)-associated syringomyelia. The findings from 15 included studies demonstrated that posterior fossa decompression, particularly when combined with duraplasty (PFDD), remains the most effective and durable treatment for restoring CSF circulation and achieving syrinx resolution. Shunting procedures, while useful in selected refractory cases, were associated with variable long-term success and higher recurrence rates. Across the reviewed studies, PFDD consistently produced marked syrinx reduction and symptomatic improvement. Several investigations documented syrinx resolution rates between 75% and 90%, confirming that restoration of CSF flow across the foramen magnum through dural expansion is the key determinant of success [1-10]. In contrast, bone-only decompression yielded lower radiologic improvement and higher recurrence, further supporting the superiority of PFDD. The introduction of arachnoid-preserving and extradural techniques was intended to reduce CSF-related complications. Maintaining arachnoid integrity was shown to minimize pseudomeningocele and CSF leakage without compromising syrinx resolution, while smaller craniectomies achieved equivalent efficacy with reduced morbidity [1-9]. Clinical improvement generally paralleled radiologic collapse, with 60-100% of patients reporting relief from headaches and sensory or motor symptoms [1-4,7]. Nevertheless, several studies noted that clinical recovery did not always correspond precisely to the degree of syrinx reduction [3-4]. In pediatric cohorts, early decompression was associated with better neurological recovery and stabilization of scoliosis, emphasizing the importance of timely intervention before irreversible spinal cord injury [7-8]. Shunting procedures were typically used as salvage therapy when decompression failed to achieve adequate syrinx resolution. In these scenarios, SSSs resulted in a 70-85% reduction in syrinx size and consistent symptomatic improvement [10-15]. However, shunt malfunction and recurrence were occasionally reported over long-term follow-up, often due to postoperative scarring or obstruction requiring revision surgery. These findings reinforce that SSS should be considered a secondary option rather than a first-line approach. CSF leakage and pseudomeningocele formation represented the most common complications, primarily in duraplasty cohorts. Extradural and arachnoid-preserving methods demonstrated lower complication rates while maintaining favorable outcomes [1-9]. Reported reoperation rates ranged from less than 10% in large multicenter PFDD series to nearly 50% in pediatric revision cases. Most reoperations effectively re-established CSF flow and achieved additional syrinx reduction [7-12]. According to the Newcastle-Ottawa Scale, 11 studies achieved high quality (scores 8/9) and four demonstrated moderate quality (scores 6-7/9), supporting the reliability of pooled findings. Despite this, heterogeneity persisted across study design, patient population, surgical technique, and follow-up duration, limiting direct quantitative comparisons. The predominance of retrospective studies and the absence of randomized controlled trials remain important limitations of the available evidence [1-15]. This review is limited by the variable quality and design of the included studies. Most were retrospective cohorts or case series with heterogeneous methodologies, sample sizes, and follow-up durations, restricting quantitative synthesis. Publication bias cannot be excluded, as studies reporting favorable outcomes are more likely to appear in the literature. Moreover, the lack of standardized definitions for clinical and radiological outcomes limited cross-study comparison. The absence of randomized controlled trials further prevents establishing a definitive causal relationship between surgical technique and outcome. Despite these limitations, the overall evidence supports PFDD as the gold-standard surgical treatment for CM-I-associated syringomyelia. Arachnoid-preserving and extradural modifications enhance safety without compromising efficacy, while syringo-subarachnoid shunting remains a valuable salvage option in cases of persistent or recurrent syrinx after adequate decompression. Early surgical intervention, particularly in pediatric patients, appears to yield superior clinical and radiological outcomes [7-9,14].

## Conclusions

PFDD provides the most consistent improvement in syrinx resolution and clinical outcomes for Chiari I-associated syringomyelia. Shunting procedures remain valuable in refractory cases but carry greater long-term risks of recurrence and malfunction. Future prospective comparative studies are warranted to refine surgical selection, minimize complications, and enhance long-term management strategies.
